# Epidemics with mutating infectivity on small-world networks

**DOI:** 10.1038/s41598-020-62597-5

**Published:** 2020-04-03

**Authors:** Sten Rüdiger, Anton Plietzsch, Francesc Sagués, Igor M. Sokolov, Jürgen Kurths

**Affiliations:** 1 Department of Physics, Humboldt-Universität zu Berlin, 12489 Berlin, Germany; 2 Potsdam Institute for Climate Impact Research (PIK), 14473 Potsdam, Germany; 3 Departament de Química Física, Universitat de Barcelona, 08028 Barcelona, Spain; 4 IRIS Adlershof, Zum Großen Windkanal 6, 12489 Berlin, Germany; 5 Saratov State University, 83, Astrakhanskaya Str., 410012 Saratov, Russia

**Keywords:** Complex networks, Nonlinear phenomena

## Abstract

Epidemics and evolution of many pathogens occur on similar timescales so that their dynamics are often entangled. Here, in a first step to study this problem theoretically, we analyze mutating pathogens spreading on simple SIR networks with grid-like connectivity. We have in mind the spatial aspect of epidemics, which often advance on transport links between hosts or groups of hosts such as cities or countries. We focus on the case of mutations that enhance an agent’s infection rate. We uncover that the small-world property, i.e., the presence of long-range connections, makes the network very vulnerable, supporting frequent supercritical mutations and bringing the network from disease extinction to full blown epidemic. For very large numbers of long-range links, however, the effect reverses and we find a reduced chance for large outbreaks. We study two cases, one with discrete number of mutational steps and one with a continuous genetic variable, and we analyze various scaling regimes. For the continuous case we derive a Fokker-Planck-like equation for the probability density and solve it for small numbers of shortcuts using the WKB approximation. Our analysis supports the claims that a potentiating mutation in the transmissibility might occur during an epidemic wave and not necessarily before its initiation.

## Introduction

In the human history, epidemic diseases such as the acquired immune deficiency syndrome (AIDS) and the pandemic influenza have killed millions of people worldwide. Recently, Influenza A (H1N1) spread in 2009, and Ebola spread in the years 2013 to 2016. Not as catastrophic as originally feared, Influenza A, which is genetically close to the Spanish flu of the early 20th century, caused 284,000 deaths^1^, while Ebola caused 11,300 deaths^2^. The Influenza epidemics, particularly, proved that a highly contagious new virus could rapidly spread to all continents of the earth in less than a year. Among animals, diseases including the African swine fever have been rapidly expanding and have required expensive steps by the responsible authorities.

To understand disease spreading and identify possible outbreak control methods, it is valuable to study contact networks and the interaction of the network structure with epidemiology^3^. The mathematical modeling of dynamical processes on networks has, in the recent years, produced an abundance of results that help to unravel the complexities of epidemic waves and predict the outcomes and risks of transmitting diseases^4–7^. Thus, the physical theory of percolation on networks^8^ was mapped to the spreading of diseases^9^. This knowledge has been used to determine transmission rates for which a disease becomes endemic^10^ by relating the critical infection rate to the threshold of percolation on networks. Moreover, effective immunization strategies to fight the spreading of a disease have been found by identifying individual nodes that should be made immune based on certain network measures^7^.

One important attribute of networks, which appears in many socio-spatial real-world networks, is the small-world property. With it, individual nodes are connected frequently in their local spatial areas^11,12^, yet they are also connected to distant nodes via a small number of intermediated nodes. Transport networks, which are common substrates for infection spreading, were found to possess the small-world property; particularly those that developed in modern times. For instance, for the spread of human diseases it may be important that individuals fly long distances by plane, which makes the human transport grid to be of small-world type^13^. Similarly, transmission networks of viruses such as the West Nile virus, which are transmitted in short-range by mosquitos and long-range by birds^14^, likely exhibit the small-world property.

Since small-world networks are so ubiquitous in our world, interest in dynamical processes for such networks has grown quickly. This particularly holds for epidemics where spatial relations between hosts (e.g., transport between villages, city, or countries) are relevant. Importantly, it was found that generally small-worldness lowers the threshold for percolation and thus also promotes the global contagion on networks with this property^5^. We here built on this earlier modeling work for epidemics on small-world networks. We assume a network of nodes which are inhabited by individuals (hosts of the pathogen). Because of transport of individuals between nodes, infecting agents can travel with them to a connected node and infect the node’s community. This process is modeled here by the established Susceptible-Infected-Recovered (SIR) model in which, for simplicity, individuals at each node are collectively in one of three states: susceptible (S), infected (I), or recovered or removed (R).

Additionally, we take into account the genetic mutations and selectivity that a spreading pathogen experiences. Viruses have very high mutation rates^15^ and several mutations can accumulate leading to substantial displacements in their genotype. In fact, mutation rates are so high that the evolution of a virus is interacting with its epidemiological behavior^16^. We here investigate the case that pathogens, by a series of random mutations, undergo changes of their genes that affect their infection or transmission probability. Such mutations of the infectivity occur for instance during adaptation to new environments or as response to antiviral or antibiotic drugs^17^. Recently, the possibility that an adaptive mutation during the West African Ebola epidemic increased transmission between humans was described^18,19^.

We specifically study the situation where the agent’s infection probability is initially subcritical (in the sense of percolation theory) but may mutate into a state with higher, supercritical infection probability. We find that in networks without small-world links, the probability of an escape to the supercritical state remains small, even if the mutation probability is large. In contrast, if small-world links are present, a supercritical mutation occurs frequently even for moderate mutation rate. The genetic change then entails a transition to a supercritical infection probability, which in turn causes a global infection of almost all nodes of the network. In contrast to the former case of epidemics assisted by small-world links, we here obtain a regime with a high chance of complete infestation of the host species due to the escape process driven by mutations. For larger numbers of long-range links, i.e., for networks approaching the global mixing of random graphs, the sweeping outbreaks disappear again.

## Mathematical Model

Our model comprises a two-dimensional spatial network of nodes that adopt one of the three SIR states. A one-dimensional fitness variable is attached to each node that hosts the virus, i.e., that is in the I-state. As motivated above, we have chosen a genetically dependent fitness variable that directly affects the node-to-node transmission probability.

**Figure 1:**
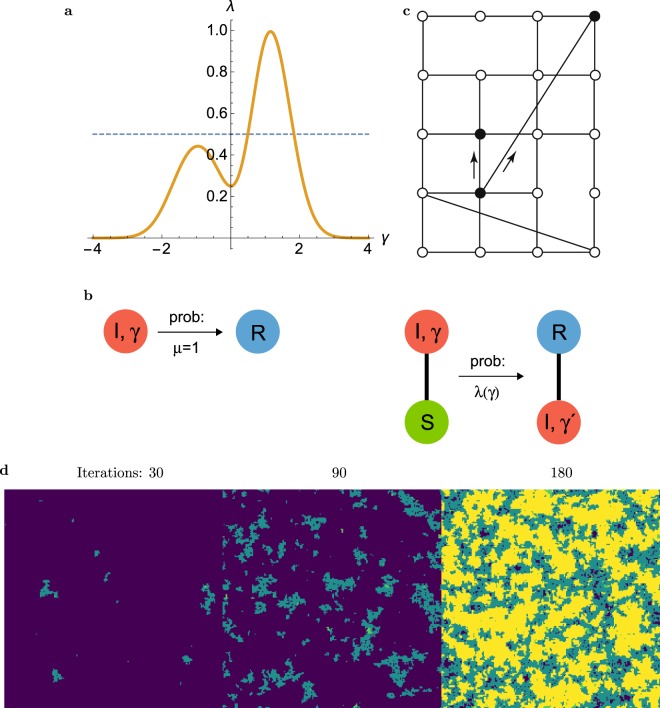
(**a**) Functional relation between the infection probability *λ* and the mutating variable *γ*. The dashed blue line shows the location of the percolation point for a square grid network of nodes. (**b**) Scheme showing the possible transitions during a time step. Every infected node recovers after a time step (left). A susceptible node that is linked to an infected node becomes infected with probability *λ*(*γ*), where *γ* is the genetic variable of the infected node. The infected node inherits the value *γ* that is then mutated to the new value $\gamma {\prime}$ in the same time step. (**c**) A two-dimensional square lattice where a fraction *p* of regular links has been replaced by long-distance links. Infections (filled circles) can travel to close neighbors but also to distant nodes. (**d**) Snapshots of the evolution of the *γ* variable on a network with 200 × 200 nodes for p = 0.01 and *χ* = 0.004 (blue - not infected, green - infected, *γ* = −1, yellow - infected with *γ* = 1). After 180 iterations the supercritical mutation covers almost all of the area.

Generally, the concept of the fitness landscape relates the genotype of an organism to its survival and reproductive success. If the fitness landscape is flat, all genotypes of a population have the same chance of replication. If however, the landscape exhibits regions of different height, with peaks surrounded by deep valleys, a population usually climbs uphill by series of small genetic changes. In our model we specifically consider the generic case where we have two peaks and one valley in the fitness space, i.e., states between the maxima are selectively disadvantageous (Fig. 1a). Thus the non-spatial part of our model is similar to point models of stochastic tunneling through a fitness valley^20–22^.

In the case of viral epidemics, the two peaks could, for example, correspond to different specified routes of transmission between individual hosts. For instance, in^23^ the possibility of an evolution to a respiratory route in mammals has been studied for the potentially dangerous A/H5N1 Influenza virus. Here, as is typical for viruses^24^, the number of genetic substitutions that are required for this virus to adapt is low (at 3 to 5). In contrast, bacteria are adapting to new environments slower, which may be linked to the fact that individual mutations have a smaller effect and many mutations may be required^25^.

Spatial effects on evolution in fitness landscapes have been considered in^26^ and were generally found to support generation of mutants even if they are less fit. This may also lead to faster adaptation in multi-peaked fitness landscapes since the mutants explore the fitness space quicker. We here propose a model where the spatial structure is more complex by allowing local spatial contacts and, additionally, far-reaching contacts.

### SIR model of epidemic spreading

The SIR model is one of the simplest models for transmission of diseases in a population of individuals. Every individual is in either one of three SIR states. The transitions between the states are

1 $$S\mathop{\to }\limits^{\lambda }I\mathop{\to }\limits^{\mu }R,$$

where *λ* and *μ* are the probabilities of infection and recovery per time step, respectively. Consider now a network of locations on which the infection spreads by specified contacts. Every node of the network contains individuals that are collectively in one of the three states. If a node is susceptible and at least one of its connected nodes is infected, it becomes infected as well with probability *λ*, whereas an infected node recovers with probability *μ*. The value of *λ* is determined by the states *γ* of the infected nodes connected to a susceptible node, see Fig. 1b. During each time step, for every S-node we calculate the probability *λ*(*γ*) for any connected I-node and let the S-node become infected according to this probability. If several connected nodes are infected, we randomly shuffle the order by which the transition is tested. Individuals in state R are immune and cannot be infected again. Note that for simplicity we consider *λ* and *μ* as probabilities for a (relatively large) discrete time step, which means that we do not obtain exponential waiting time distribution for the transitions.

We initially start with a network of susceptible nodes and a single randomly chosen infected site. After a certain time, the system reaches a stable equilibrium where no infected nodes remain. The fraction of recovered nodes depends on the infection and recovery probabilities *λ* and *μ* as well as on the topology of the network. For simplicity, we set the recovery probability to *μ* = 1 so that an infected node is recovered or removed after one time step.

### SIR Model in a Watts-Strogatz-Network

To take into account the geographical space om which the disease spreads, we consider a two-dimensional regular square lattice with *N* nodes and periodic boundary conditions. In such a grid every node has four nearest neighbors and the disease would spread spatially like a wave front. It is known that, in analogy to the problem of percolation on lattices^27^, for a supercritical infection probability *λ* > 0.5 a nonzero probability for the spreading of the infection exists^28^.

Next, every link is rewired to a random node with probability *p* which generates shortcuts and decreases the average path length. The resulting network is a generalized Watts-Strogatz-Graph^29^ with mean degree *k* = 4 (Fig. 1c). For very large *p* close to 1 we obtain a well-mixed random network. It was shown in^5^ that rewiring reduces the threshold of percolation in the network so that the critical *λ* is smaller than 0.5.

### Mutations of the Infection Probability

In many investigations of SIR on networks, the infection probability *λ* is a constant of the dynamical process. Now, incorporating genetic adaptation, we assume that the infection probability can be varied by mutations. The disease could for instance be a virus that spreads from node to node on the network and can undergo mutations of its genome.

Here we assume that the individuals at each node are collectively at the same genetic state. This may appear unrealistic at first; however, it depends indeed on the respective time scales of mutations, fixations and transmissions to determine how close a population is to this scenario. Given the phylogenetic data for instance for the case of Ebola epidemic in 2013-2016, it appears that the genetic variability of the entire virus population of the outbreak is much larger than the variability at each infection site or country^30^. Following this line of thought we consider the limit in which establishment of a strain at each node occurs quickly, so that the local population is collectively characterized by a single virus strain. We will study two scenarios, one for pathogens requiring a small number of mutational steps for adaptation and one for pathogens requiring a large number of steps, where a continuous approximation may be valid:

i) In a first case, we introduce a relation *λ*_d_(*γ*) with the mutating genetic variable *γ*, which can have three values: -1 in the initial state (no mutation), 0 in a neutral state (one mutation), 1 in the supercritical state (two mutations). The transmission probability *λ*_d_ depends on *γ* as

**Table:** 

*γ*	−1	0	1
*λ*_d_	0.45	0.35	0.95

This mapping is chosen so that it has two maxima of optimal fitness. One peak is set slightly below the critical infection probability of 0.5 so that the epidemic wave typically covers at most a small finite domain. A second peak is chosen well above the critical infection probability corresponding to a well adapted genotype of the species that may typically lead to a global epidemic wave. In keeping with the valley crossing mentioned above, the intermediate state *γ* = 0 presents a minimum in the fitness, with which many of the nodes do not pass on the pathogen. As an initial condition we choose *γ* = −1.0 at all network nodes and initially infect one randomly chosen node. Then the infection probability is initially below the critical value at *λ* = 0.45. If a node *i* is in state *I*, the sequence variable *γ*_*i*_ mutates in each iteration step with the rates given by the scheme

2 $$-1\underset{{\rm{\chi /2}}}{\overset{{\rm{\chi }}}{\rightleftarrows }}0\underset{{\rm{\chi }}}{\overset{{\rm{\chi /2}}}{\rightleftarrows }}1$$

where the -1, 0, 1 are the state values of *γ*. The parameter *χ* can be interpreted as a mutation rate parameter that quantifies the probability per time step of the respective transitions. The transmission probability *λ*(*γ*_*i*_) changes according to the table above. If a node *i* infects one of its connected sites, the genetic variable *γ*_*i*_ is inherited. This value is then, during the same iteration step, mutated. After an infected node transitions to the R-state, its *γ*_*i*_ value remains unchanged.

ii) In a second scenario we assume a continuous variable *γ* and a corresponding function *λ*_c_(*γ*), see Fig. 1a. In the literature on evolutionary dynamics, *γ* is called the sequence variable and the function *λ* can be viewed as the fitness landscape in the sequence space^31,32^. Usually, the sequence space is high-dimensional, but for simplicity we consider here only a one-dimensional one.

## Results

### Discrete case

Given the initial infected site and its infection probability *λ*, an infection can generally spread through parts of the network. If *λ* stays constant, there is a chance that the pathogen infects small or large spatial domains, which can be mathematically described by the established percolation theory. However, as a consequence of the mutations, the infection probability changes and enables more substantial evolutions of the epidemic. If, in this process, the genetic variable *γ* evolves towards 1, the epidemic may be able to spread throughout the entire network since *λ* is then larger than the critical value of 0.5. Fig. 1d shows an exemplary evolution of the genetic variable *γ* where most of the network is eventually infected by the mutated pathogen (green and yellow).

To quantify the effect, we let the system evolve for 10^3^ to 10^6^ timesteps for each parameter set. For each run we generate a new network with the given parameters. We stop a simulation when the number of R nodes does not change anymore. We then determine the fraction of those runs in which the R-state *covers more than* 90% of the network, i.e., the outbreak leads to almost complete coverage. This event will be called a sweep in the following.

**Figure 2:**
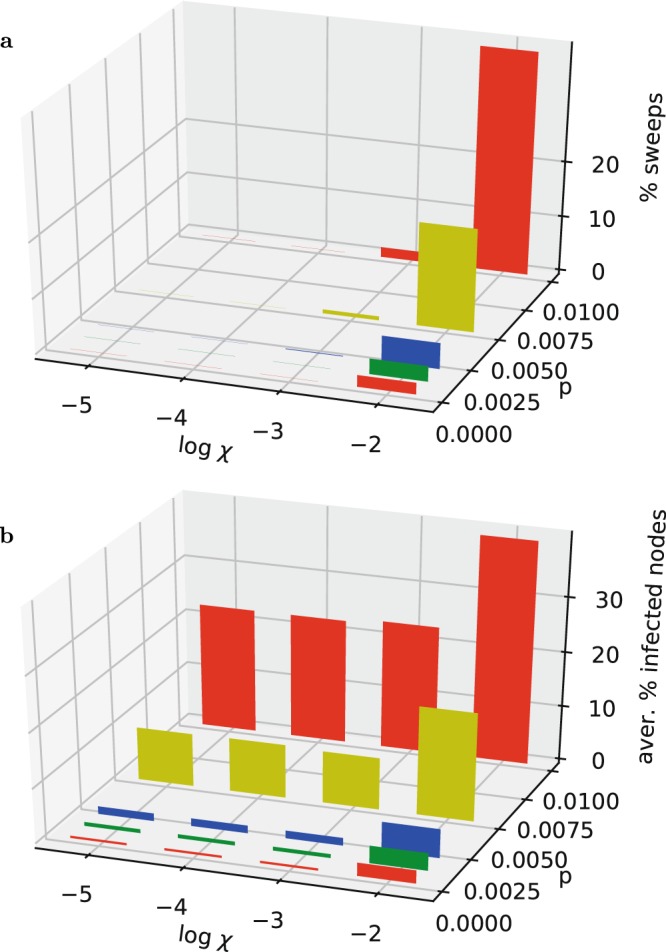
Epidemics of the SIR model with discrete mutations of the infection probability *λ*_d_(*γ*) in a square lattice (*N* = 200 × 200) and a Watts-Strogatz-graph – dependence on two parameters: mutation rate *χ* and rewiring fraction *p*. (**a**) The z-axis is the share of runs in which almost complete coverage of the graph is obtained (number of R-nodes is larger than 90% at final state). (**b**) Share of R nodes averaged for 10,000 runs for each parameter set.

We find that the capability to sweep the network depends strongly on the structure of the network (Fig. 2a). If no or few long-range links are present and the genetic mutation rate is weak or moderate, the infection wave quickly dies out and overall infection is rare. However, our simulations reveal that for a rewiring rate *p* > 0.005 a moderate mutation rate suffices to obtain a large probability of full epidemics. In these epidemics almost all nodes of the network become infected.

Note that this does not mean that there are no infections spreading for small rates or without mutations. In fact, without mutations (i.e., for a constant infection probability *λ* ≈ 0.45) infections can spread. This can happen if, as was mentioned above, shortcuts enable the spreading of infections even if the *λ* value predicts a subcritical behavior for a pure grid. To discuss this in detail we now determine the share of infected nodes per run and average it over all runs. Fig. 2b shows that the average number of infected nodes is substantially larger than 0 for *p* > 0.005 and all mutation rates. It contrast to the fraction of sweeps, it does not vanish with decreasing *χ*.

**Figure 3:**
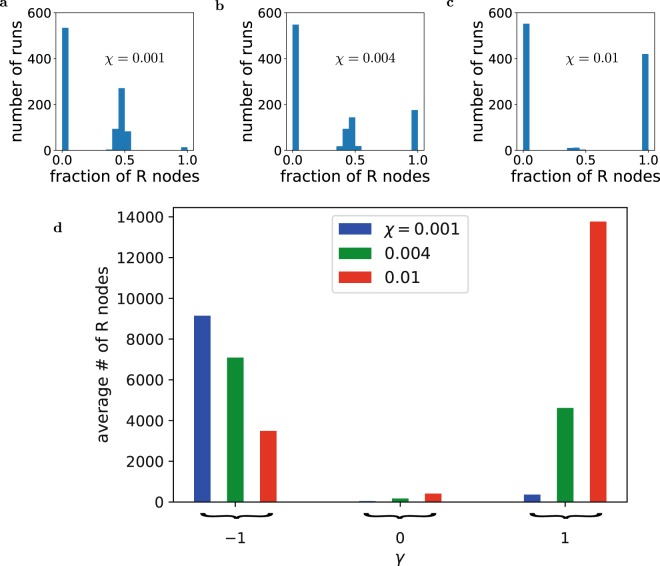
(**a**–**c**) Histograms showing the distribution of the number of R nodes in the final steady state per run for a total of 1,000 runs: *χ* = 0.001 (**a**), 0.004 (**b**), 0.01 (**c**). (**d**) Histogram of the *γ* variable (−1 or 1, no nodes reside in the 0 state in the final configuration) for the three values of mutation rate *χ* (*p* = 0.01).

The difference of our scenario with mutations to this former mutationless case of SIR in small world networks is the generation of an explosive giant component in a fraction of the runs. For the usual percolation, close to the transition point, the size of a giant component is smaller than the total population. Accordingly, for small mutation rate it is found that the distribution of the fraction of infected nodes per run has a peak at a value between 0 and 1, see Fig. 3a. This means that in a given run the infection either hits the giant component at, e.g., 50% of all nodes, or the contagion dies out. However, for sufficiently frequent mutations, in each of our small world networks there is either an almost complete coverage (>90%) or only a tiny fraction of the nodes becomes infected and removed, see Fig. 3c.

This points to a very distinctive feature of epidemic outbreaks with critical mutations: If the average fraction of infected nodes is, e.g., 30%, it could either mean that in each run around 30% of the nodes are infected, or, in an extreme case, in 30% of the runs almost all nodes are infected, while in 70% of the runs most nodes stay healthy. From a public health point of view we think that the second case is the more alarming one, since an outbreak of a deadly virus could lead to extinction of the entire population. Therefore, in our model, with short-cuts and random mutations, the outcomes and assessments of risks are different exactly in this respect that the chance of extinction rises dramatically.

The reason for this different behavior in our mutating SIR model is the fact that for sufficiently large mutation rate the sequence variable *γ* can become 1 and settle at the global extremum of the fitness landscape. Fig. 3d demonstrates this by comparing a case of small *χ* (blue bar), moderate *χ* (green) and large *χ* (red). Only in the last two cases, we obtain escape from the fitness maximum at *γ* = −1.

**Figure 4:**
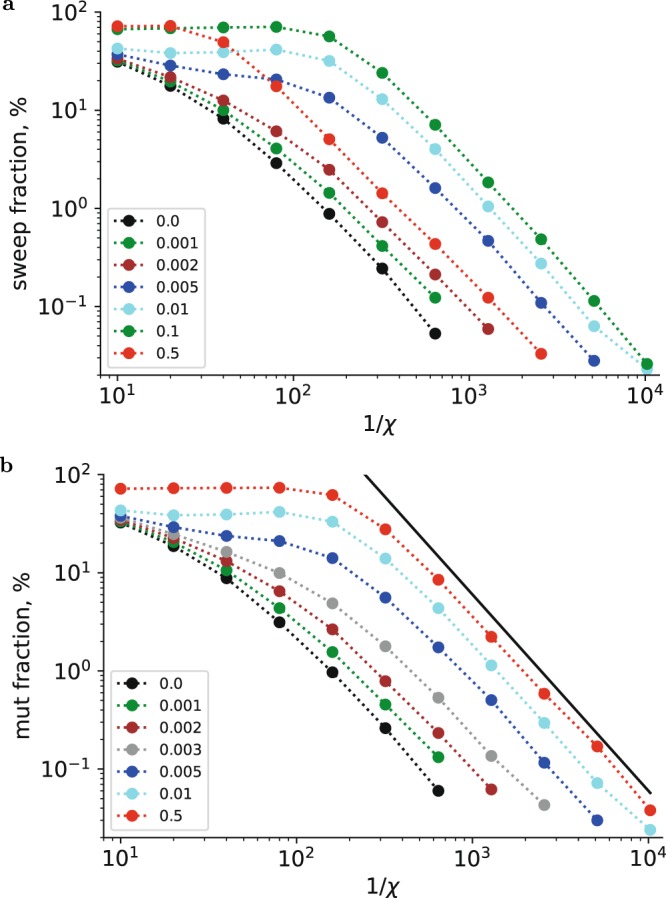
Discrete mutation model. (**a**) Percentage of sweeps in its dependence on *χ* (for indicated *p* values). (**b**) The same plot for the mutation probability. The solid line shows the slope of -2 for small *χ*.

To understand the causes of the described phenomenon, we now analyze the scaling behavior of the percentage of sweeps in dependence on the mutation rate, *χ*, and the fraction of shortcuts, *p*. Fig. 4a presents a double-logarithmic plot in which the sweep fraction is plotted versus 1∕*χ*. There is a clear linear relation on the log-log scales for small *χ*, which is characterized by a slope independent on *p*. With increasing *p*, the lines are shifted to larger values in sweep fraction. For larger mutation rates, the linear dependence is lost and the fraction of sweeps saturates for all *p*. For very large *p*, we note that the trend reverses and much a smaller sweep fraction results for a large range in *χ* (red curve, *p* = 0.5).

We will now present a theoretical explanation for the observed scaling behavior. This is made easier by splitting the phenomenology of a sweep into occurrence of a critical mutation and a following epidemic wave. We now only consider the probability that during a run at least one critical mutation occurs. Plots of this probability (i.e., the probability that during a run at least one node reaches *γ* ≥ 0) appear similar to those for the sweep fraction for small *p*, see Fig. 4b. For small *χ* the slopes of the curves apparently approach -2 (see solid black line for comparison).

This quadratic dependence on *χ* can be reasoned in the following way: The probability that at least one of the sites mutates twice to reach *γ* = 1 is

3 $${P}_{{\rm{mut}}}=1-{\left(1-\frac{{\chi }^{2}}{2}\right)}^{s}=1-{e}^{s{\rm{ln}}(1-{\chi }^{2}/2)}\approx 1-{e}^{-s{\chi }^{2}/2},$$

where *s* is the number of sites belonging to the finite cluster activated by the first infected node. The size of the cluster is distributed according to *n*(*s*) ~ *s*^−*τ*^ with a universal scaling exponent *τ* ≈ 2.05^33^. For very small *χ* we can further approximate *P*_mut_ ≈ *sχ*^2^∕2 and we obtain the mean probability for at least one supercritical mutation:

4 $${P}_{{\rm{mut}}}\approx \mathop{\sum }\limits_{s=1}^{\infty }sn(s)\frac{s{\chi }^{2}}{2} \sim \frac{{\chi }^{2}}{2}\mathop{\sum }\limits_{s=1}^{\infty }{s}^{2-\tau }.$$

This scaling with *χ* is the one observed in Fig. 4b (solid black line) for small rates. For larger rates we can use the approximation in Eq. (3) and evaluate the sum up to a cut-off:

5 $${P}_{{\rm{m}}{\rm{u}}{\rm{t}}}\sim \mathop{\sum }\limits_{s=1}^{{\rm{\infty }}}sn(s)(1-{e}^{-s{\chi }^{2}/2})$$

6 $$\approx (2-\tau ){\int }_{2/{\chi }^{2}}^{{\rm{\infty }}}{s}^{1-\tau }{\rm{d}}s$$

7 $$={\left(\frac{2}{{\chi }^{2}}\right)}^{2-\tau }.$$

Since 4 − 2*τ* ≈ − 0.1 we expect a very weak dependence for larger *χ*, which is indeed the case in our simulation data. The fact that the percolation behavior predicts the *χ* dependence of the mutation fraction suggests that the increase of *P*_mut_ with *p* is driven by the percolation transition.

Finally, discussing the probability of a sweep following a mutation, Fig. 4a shows that the fraction of sweeps declines again for *p* larger than 0.1 even if the number of critical mutations does not shrink. An obvious scenario is that for large *p* the giant cluster formed by the non-mutated pathogens (*γ* = −1) is large and spanning the entire domain quick enough so that much of the network is sufficiently covered with the subcritical infection before the supercritical form can spread. This would particularly effect those cases in which the mutation rate is small, such that a critical mutation needs much time to develop.

### Continuous case

For this case, we introduce a functional relation *λ*(*γ*) with the continuous mutating genetic variable *γ*:

8 $$\lambda (\gamma )=\exp (-{\gamma }^{2})(0.55{\gamma }^{4}+0.7{\gamma }^{3}+1.1{\gamma }^{2}+0.25).$$

This exemplary function is shown in Fig. 1a and is chosen so that it has two maxima, one below and one above the critical infection probability.

As before, we start with *γ* = −1.0 at all network nodes and initially infect one randomly chosen node. If a node *i* is in state *I*, the sequence variable *γ*_*i*_ mutates in each iteration step by

9 $${\gamma }_{i}\to {\gamma }_{i}+{\xi }_{i}$$

where *ξ*_*i*_ is a Gaussian variable with mean zero and standard deviation *σ*.

**Figure 5:**
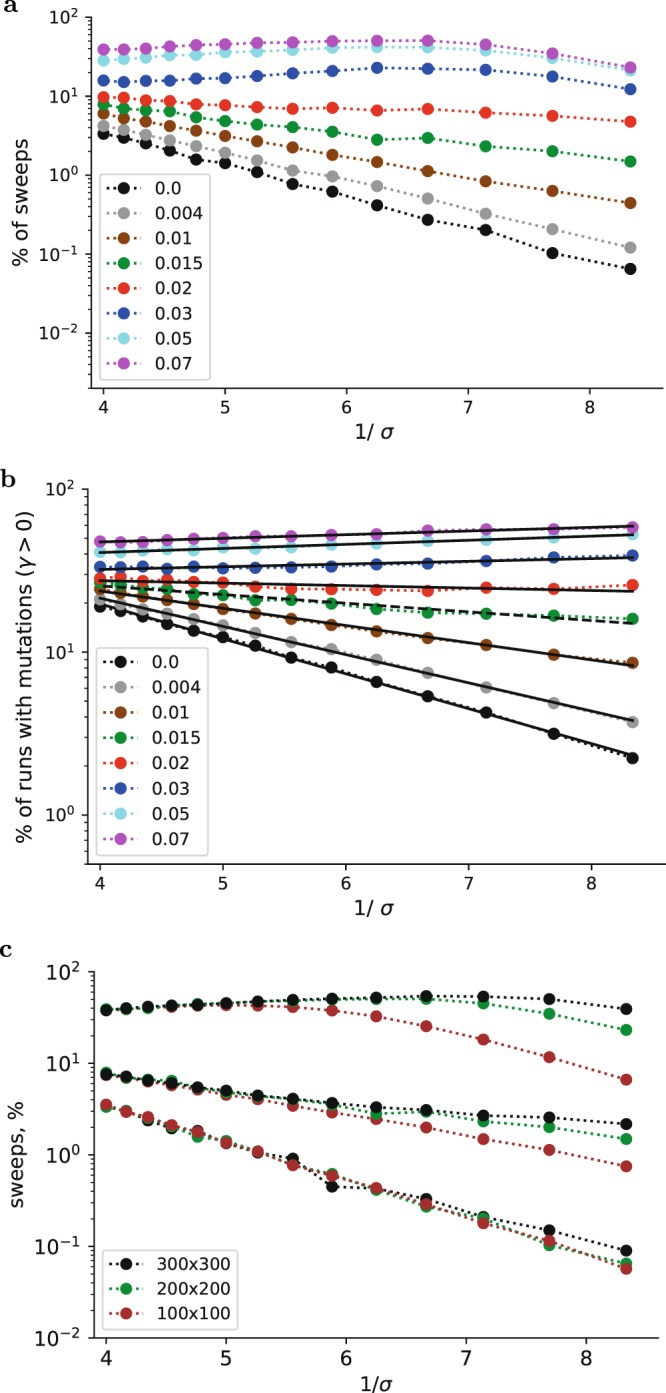
Continuous mutation model. (**a**) Percentage of sweeps in its dependence on *σ* (for indicated *p* values), continuous case. (**b**) The same plot for the mutation probability. Dashed lines are linear fits. (**c**) A finite-size effect is visible at large *p* = 0.07 comparing a lattice of 300 by 300 points (black) compared to 200 by 200 points (green) and 100 by 100 points (brown) (for *p* = 0, 0.015 and 0.07, from bottom to top).

At first glance, we find a similar qualitative behavior as in the discrete case: sweeps in general occur for larger \sigma and *p* values, see Fig. 5a. The mutation probability scales now exponentially with several regimes. For very small noise, the mutation probability disappears exponentially with *σ* (data not shown). Similar to the discrete case, the slopes of these graphs do not depend on *p* in this regime.

For larger noise, the scaling depends on the fraction of long bonds. Fig. 5a presents the logarithm of the sweep fraction plotted versus the inverse of *σ*. There appears an exponential relation for a range of approximately 4 < 1∕*σ* < 7 with the slopes depending on *p*. For larger rewiring probabilities *p* there emerges a slight kink for higher values of 1∕*σ*. This effect is due to the finite grid size. Simulations with lattices of different sizes show that the large-noise regime extends to higher 1∕*σ* for larger lattices (Fig. 5c). Results for lattice sizes 200 × 200 and 300 × 300 agree for 1∕*σ* values up to 7, which also appear to limit the range of linear fitting.

Plots of the mutation probability *P*_mut_ exhibit exponential scaling, see Fig. 5b. Since we discovered a scaling different from the discrete case, an explanation of the phenomenon should be based on a more complex model. We will approximate the active cluster as a regular tree with a coordination number *C* (the number of subbranches attached to each node). The evolution of the distribution of *γ* values on the tree for a given time can then be approximated by the birth-death equation

10 $$\frac{\partial }{\partial t}P={\sigma }^{2}\frac{{\partial }^{2}}{\partial {\gamma }^{2}}P+[C\lambda (\gamma )-1]P$$

with *P* = *P*(*γ*, *t*). The expression in the square brackets denotes the local proliferation rate. As shown in the SI Appendix 1, the total probability *s* of escaping through the value of *γ* = 0 can be obtained by means of a Wentzel-Kramers-Brillouin (WKB) approximation

11 $${\rm{\log }}\,s \sim -\frac{1}{\sigma }{\int }_{-1+\epsilon }^{0}\sqrt{r(x)}dx$$

with *r*(*γ*) = 1 − *Cλ*(*γ*) being the local decay rate. We recover the observed 1∕*σ* dependence with the slope ${\int }_{-1+\epsilon }^{0}\sqrt{r(x)}dx$. For small *p* the coordination number *C* can be determined by equating the integral to the slopes in Fig. 5b. We obtain, for instance, *C* = 2.06 for *p* = 0.

Our numerical simulations of Eq. (10) showed that the exponential scaling found in the WKB approximation survives for larger *C* values (data not shown) even if the corresponding physically relevant WKB solution cannot be found. Thus, the model tree network can give a good explanation for the exponential scaling with mutation rate *σ*.

A few words on the limitations of this approximation are in order. The dependence of *C* on the network properties or, in our case, on *p*, however, remains phenomenological. Additional numerical analysis shows that the described prevalence of supercritical mutations for larger *p* does not occur in a grid-like network. SI Appendix, Fig. S1 shows that the probability for supercritical mutations is much smaller for a local network (without short-cuts) even if the mean size of the infected clusters is similarly large as in the small-world case. This suggests that increasing the number of long links in the network helps the mutations by way of (i) increasing the percolation cluster (i.e., lowering the percolation threshold) and (ii) by lifting local blocking of mutated nodes. Thus, we conclude that, besides the increasing percolation cluster, it is the topology of the small-world network that drives the occurrence of mutations and global epidemics in our continuous model.

## Conclusions

We have shown that the interaction of epidemic transmissions of a mutating pathogen with the structure of a network can produce a transition to a regime of complete infection of a host species spatially distributed on the network. The problem has been framed in terms of stochastic escape from a subcritical transmission probability to a supercritical transmission probability. The escape to the potentially disastrous supercritical regime is facilitated by a small number of long-range links on the spatial grid of hosts. We are thus closing a loop in which transmission enables mutation and mutation enables the spreading of disease on the complete network.

Our work suggests that risk assessment should be substantially different depending on whether or not mutations are occurring. Under noisy conditions (i.e., allowing mutations) infections are not extensively worse (in a statistical sense) compared to noise-free condition, as the average percentages of infected sites do not change substantially. But mutations are potentially risky, since they are likely to produce a global outbreak in a given population, which is explosive and fast. This makes the latter situation far more difficult and challenging to control epidemiologically.

The effect that we have described rests qualitatively on two aspects. First, the small-world links drive the network close to or beyond the critical point for percolation. Thus, for *p* large enough, the infected node often initiates a large cluster, where each node can undergo a mutation and the total probability for a supercritical mutation is high.

Second, the long-range connections allow the mutated form to emerge and to move freely to uninfected parts of the network, so that the mutation can quickly spread and cover large parts of the network (sweep). The latter, however, only holds if the original type has not covered the entire domain before the mutated type appears. Typically, if *p* is large, the speed of an epidemic wave is increased. In this situation, the non-mutated type indeed covers large parts of the network before the supercritical pathogens can propagate. Oftentimes, in this situation the non-mutated form remains sparse while covering the network so that the probability of full blown epidemics is much reduced, see Figs. 4a,b.

Qualitatively the behavior we describe is similar for the cases of discrete and continuous mutational spaces. The scalings of mutation probabilities, however, are different for the two cases. While in the discrete case the probability to a supercritical mutant state scales with the mutation rate in power law form, we found exponential behavior for the continuous case. For both models, we presented analytical derivations of these relations. The scalings with the small-worldness parameter *p*, however, are more complex and our discussion in Appendix S2 gives a first hint on the differences of the continuous and discrete cases. For the limit of a continuous mutation space, our simulations suggest that long-range links additionally help to overcome a local blocking to the spreading of pathogens in intermediate genetic states (see Appendix S2 for more details).

To fend off disease spreading, our results suggest several approaches. First, in the early stage of the epidemics, one should prevent the critical mutation. This could be achieved by a suppression of long-range links for instance by shutdown of transport hubs or preferential vaccination of regions around long-range connected nodes. In this way the development of isolated regions of the network, where the mutation can proceed without competition, is diminished. Second, after a supercritical pathogen has established, one needs to prevent spreading by removal of links around the supercritical nucleation site. Third, it is also conceivable, that, depending on the topology, an increase of *p* can result in the spreading of the non-mutated form, thus preventing the infection by the mutated form. This and other questions regarding, for instance, the influence of vaccinations are subject of current work on the problem.

It is interesting to compare our results with the recent debate whether a potentiating mutation could occur during an epidemic or whether it usually occurs before the start of the epidemic^34^. For instance, in the case of the Ebola outbreak 2013-2016 it was suggested that one or several mutations identified during the outbreak caused the strong infectivity of the virus^18,19^. Potentially this question might also determine the chances of a severe mutation of the Ebola virus from a droplet to an airborne transmission route^35^. It has been argued that within-host selectivity provides a much quicker adaptation than between-host selectivity since the time-scale of selectivity is much shorter within a host than between hosts. In the context of our study, the within-host scenario is one of well-mixed dynamics (large *p*, see Fig. 4a), where we found a domination of the non-mutated form. Only for intermediate small-worldness do we find the mutated form to be competitive. Our results suggest that the decisive mutation benefits from the small-worldness and may thus arise during the epidemic wave on a network with the suitable properties. This finding substantiates the claims for a mutation during the epidemic^18,19^.

Finally, the relevance of our work for other network types should be studied in the future. Besides the small-worldness, other topological properties as scale-freeness and degree correlation have been shown to play an important role for epidemic spreading^36,37^ and should therefore be taken into account. We expect that a similar mechanism to the one described here for the case of epidemics may also be relevant for the related problems of spreading on networks of other phenomena such as rumors and influences.

## Supplementary information

Supplement file.

## References

[1] Dawood FS (2012). Estimated global mortality associated with the first 12 months of 2009 pandemic influenza A H1N1 virus circulation: a modelling study. The Lancet infectious diseases.

[2] *WHO: Ebola situation report 30 March 2016* (2016).

[3] Danon, L. *et al*. Networks and the epidemiology of infectious disease. **2011** (2011). 10.1155/2011/284909 PMC3062985 21437001

[4] Moore C, Newman ME (2000). Epidemics and percolation in small-world networks. Physical Review E. 61.

[5] Sander L, Warren C, Sokolov I, Simon C, Koopman J (2002). Percolation on heterogeneous networks as a model for epidemics. Mathematical Biosciences.

[6] Colizza V, Pastor-Satorras R, Vespignani A (2007). Reaction-diffusion processes and metapopulation models in heterogeneous networks. Nature Physics.

[7] Pastor-Satorras R, Castellano C, Van Mieghem P, Vespignani A (2015). Epidemic processes in complex networks. Reviews of Modern Physics.

[8] R. Cohen & S. Havlin, *Complex networks: structure, robustness and function* (Cambridge university press, 2010).

[9] Wang W, Tang M, Stanley HE, Braunstein LA (2017). Unification of theoretical approaches for epidemic spreading on complex networks. Reports on Progress in Physics.

[10] M. Newman, *Networks: an introduction* (Oxford university press, 2010).

[11] Barthélemy M (2011). Spatial networks. Physics Reports.

[12] Sun G-Q, Jusup M, Jin Z, Wang Y, Wang Z (2016). Pattern transitions in spatial epidemics: Mechanisms and emergent properties. Physics of Life Reviews.

[13] Amaral LAN, Scala A, Barthelemy M, Stanley HE (2000). Classes of small-world networks. Proceedings of the National Academy of Sciences.

[14] Sule WF (2018). Epidemiology and ecology of West Nile virus in sub-Saharan Africa. Parasites & Vectors.

[15] Duffy S (2018). Why are RNA virus mutation rates so damn high?. PLoS Biology.

[16] Pybus OG, Rambaut A (2009). Evolutionary analysis of the dynamics of viral infectious disease. Nature Reviews Genetics.

[17] Pybus OG, Tatem AJ, Lemey P (2015). Virus evolution and transmission in an ever more connected world. Proceedings of the Royal Society B: Biological Sciences.

[18] Diehl WE (2016). Ebola virus glycoprotein with increased infectivity dominated the 2013–2016 epidemic. Cell.

[19] Urbanowicz RA (2016). Human adaptation of Ebola virus during the West African outbreak. Cell.

[20] Iwasa Y, Michor F, Nowak MA (2004). Stochastic tunnels in evolutionary dynamics. Genetics.

[21] Weinreich DM, Chao L (2005). Rapid evolutionary escape by large populations from local fitness peaks is likely in nature. Evolution.

[22] Altland A, Fischer A, Krug J, Szendro IG (2011). Rare events in population genetics: stochastic tunneling in a two-locus model with recombination. Physical Review Letters.

[23] Russell CA (2012). The potential for respiratory droplet-transmissible A/H5N1 influenza virus to evolve in a mammalian host. Science.

[24] Longdon B, Brockhurst MA, Russell CA, Welch JJ, Jiggins FM (2014). The evolution and genetics of virus host shifts. PLoS Pathogens.

[25] Bonneaud C, Weinert LA, Kuijper B (2019). Understanding the emergence of bacterial pathogens in novel hosts. Philosophical Transactions of the Royal Society B.

[26] Cooper JD, Neuhauser C, Dean AM, Kerr B (2015). Tipping the mutation-selection balance: Limited migration increases the frequency of deleterious mutants. Journal of Theoretical Biology.

[27] A. Bunde & Havlin S. Percolation I in *Fractals and Disordered Systems* (Springer, 1996), pp. 59–114.

[28] Newman ME (2002). Spread of epidemic disease on networks. Physical Review E.

[29] Watts DJ, Strogatz SH (1998). Collective dynamics of small-world networks. Nature.

[30] Holmes EC, Dudas G, Rambaut A, Andersen KG (2016). The evolution of Ebola virus: Insights from the 2013–2016 epidemic. Nature.

[31] M. A. Nowak, *Evolutionary dynamics* (Harvard University Press, 2006).

[32] De Visser JAG, Krug J (2014). Empirical fitness landscapes and the predictability of evolution. Nature Reviews Genetics.

[33] D. Stauffer & A. Aharony, *Introduction to percolation theory* (Taylor & Francis, 2014).

[34] Bedford T, Malik HS (2016). Did a single amino acid change make Ebola virus more virulent?. Cell.

[35] Judson S, Prescott J, Munster V (2015). Understanding ebola virus transmission. Viruses.

[36] May RM, Lloyd AL (2001). Infection dynamics on scale-free networks. Physical Review E.

[37] Wang Y, Ma J, Cao J, Li L (2018). Edge-based epidemic spreading in degree-correlated complex networks. Journal of Theoretical Biology.

