# Supplementary material for: Epidemics with mutating infectivity on small-world networks
Source: Sci Rep. 2020 Apr 3;10:5919. doi: 10.1038/s41598-020-62597-5 (PMC7125191; doi:10.1038/s41598-020-62597-5)
Supplement: Supplementary file 1 — Supplement file. [file 41598_2020_62597_MOESM1_ESM.pdf]

EPIDEMICS WITH MUTATING INFECTIVITY ON SMALL-WORLD NETWORKS
SI APPENDIX

STEN RÜDIGER, ANTON PLIETZSCH, FRANCESC SAGUÉS, IGOR M. SOKOLOV, JÜRGEN KURTHS

SI Appendix 1: Analytical solution by WKB method

Deriving the stationary equation. We start by the discussion of the mean-field birth-death equation

$$\frac{\partial}{\partial t}p = \sigma^2 \frac{\partial^2}{\partial \gamma^2}p + [C\lambda(\gamma) - 1]p \tag{1}$$

with $p = p(\gamma, t)$, where the expression in the square brackets denotes the local proliferation rate. The initial condition to this equation is $p(\gamma, 0) = \delta(\gamma + 1)$.

We are interested in the total probability of achieving the value of $\gamma = 0$, which is defined as $s(\sigma) = -\sigma^2 \int_0^\infty \left.\frac{\partial p}{\partial \gamma}\right|_{\gamma=0} dt$ at the absorbing boundary $\gamma = 0$ where p is set to zero. In the diffusion picture this corresponds to a situation that a particle starting at $\gamma = -1$ achieves in the diffusion motion the border of a semi-infinite interval without having being killed underway.

To simplify the discussion, we can use the approach similar to the flow-over-population approach for calculating the mean first passage time for the diffusive process in an external potential. We now consider a situation when particles are continuously introduced at $\gamma = -1$ by an external source creating a particle flux $j = 1$ (i.e. one particle is introduced on the average per unit time). In the stationary situation then s will be the number of particles leaving the interval through its right border per unit time. The value of s expressed via this stationary solution $n(\gamma)$, i.e. the solution of the equation

$$\sigma^2 \frac{\partial^2}{\partial \gamma^2}n + [C\lambda(\gamma) - 1]n + j\delta(\gamma + 1) = 0,$$

and will read

$$s = -j^{-1}\sigma^2 \left.\frac{\partial n}{\partial \gamma}\right|_{\gamma=0}. \tag{2}$$

To see this we note that the solution of the equation Eq.(1) with the given concentrated initial condition is essentially the Green's function of Eq.(1), i.e. the solution of

$$\frac{\partial}{\partial t}G(\gamma, t|\gamma', t_0) - \sigma^2 \frac{\partial^2}{\partial \gamma^2}G(\gamma, t|\gamma', t_0) - [C\lambda(\gamma) - 1]G(\gamma, t|\gamma', t_0) = \delta(\gamma - \gamma')\delta(t - t_0)$$

with $\gamma' = -1$, with the corresponding boundary condition $G(0, t|\gamma', t_0) = 0$ for all $-\infty < \gamma' \leq 0$. Due to the time-homogeneity this Green's function depends only on $\tau = t - t_0$ so that $G(\gamma, t|\gamma', t_0) = G(\gamma, t - t_0|\gamma')$. Now our s reads

$$s = -\sigma^2 \int_0^\infty \frac{\partial}{\partial \gamma} G(\gamma, t| - 1)\bigg|_{\gamma=0} dt = -\sigma^2 \frac{\partial}{\partial \gamma} \int_0^\infty G(\gamma, t| - 1)|_{\gamma=0}\, dt. \tag{3}$$

The solution $n(\gamma, t)$ for the density in presence of a general source $J(\gamma, t)$ switched on at $t = 0$, i.e. the solution of the equation

$$\frac{\partial}{\partial t} n(\gamma, t) = \sigma^2 \frac{\partial^2}{\partial \gamma^2} n(\gamma, t) + [C\lambda(\gamma) - 1] n(\gamma, t) = J(\gamma, t)$$

is then given by

$$n(\gamma, t) = \int_{-\infty}^0 d\gamma' \int_0^t G(\gamma, t - t'|\gamma') J(\gamma', t') dt'.$$

Let us now take $J(\gamma, t) = j\delta(\gamma + 1)\Theta(t)$ with constant j, and assume that the stationary solution establishes at long times. In this case this solution is

$$n(\gamma) = j \int_0^\infty G(\gamma, t''| - 1) dt''.$$

Comparing this with Eq.(3) we get Eq.(2).

Solution by WKB approximation. Let us first assume the situation to be such that the proliferation rate is negative everywhere inside the interval (which is the case in all situations where the epidemic propagation without mutations is impossible). Now our equation has the form

$$\sigma^2 \frac{\partial^2}{\partial \gamma^2} p + [C\lambda(\gamma) - 1] p = -j\delta(\gamma + 1) \tag{4}$$

with the boundary condition $p(0) = 0$. The corresponding equation right and left from the injection point is homogeneous

$$\sigma^2 \frac{\partial^2}{\partial \gamma^2} p - r(\gamma) p = 0$$

with $r(\gamma) = 1 - C\lambda(\gamma)$ being the local decay rate, and its solution for small σ will be faithfully reproduced by the WKB approximation:

$$p^+(\gamma) = c_1 r^{-\frac{1}{4}}(\gamma) \exp\left(\frac{1}{\sigma} \int_{-1+\epsilon}^\gamma \sqrt{r(x)} dx\right) + c_2 r^{-\frac{1}{4}}(\gamma) \exp\left(-\frac{1}{\sigma} \int_{-1+\epsilon}^\gamma \sqrt{r(x)} dx\right),$$

where the two integration constants have to be set to fulfill the matching condition in the injection point and the boundary condition at 0. To fulfill the last one we have to set

$$c_1 = -c_2 \exp\left(-\frac{2}{\sigma} \int_{-1+\epsilon}^0 \sqrt{r(x)} dx\right).$$

Now only one integration constant ($c = c_2$) is free:

$$\begin{aligned} p^+(\gamma) &= cr^{-\frac{1}{4}}(\gamma)\left[\exp\left(-\frac{1}{\sigma}\int_{-1+\epsilon}^{\gamma}\sqrt{r(x)}dx\right)\right. \\ &\left. - \exp\left(-\frac{2}{\sigma}\int_{-1+\epsilon}^{0}\sqrt{r(x)}dx\right)\exp\left(\frac{1}{\sigma}\int_{-1+\epsilon}^{\gamma}\sqrt{r(x)}dx\right)\right]. \end{aligned}$$

The derivative of this expression at $\gamma = 0$ is

$$\left.\frac{\partial p^+}{\partial \gamma}\right|_{\gamma=0} = -2cr^{\frac{1}{4}}(0)\sigma^{-1}\exp\left(-\frac{1}{\sigma}\int_{-1+\epsilon}^{0}\sqrt{r(x)}dx\right).$$

(note that the square bracket in the previous expression vanishes at $\gamma = 0$!) The constant c can be obtained from matching conditions at the injection point $\gamma = -1$. The solution of our equation left from the injection point contains only growing exponent due to the boundary condition at $-\infty$:

$$p^-(\gamma) = c_3 r^{-\frac{1}{4}}(\gamma)\exp\left(\frac{1}{\sigma}\int_{-1}^{\gamma}\sqrt{r(x)}dx\right).$$

Matching the $p^+(-1)$ and $p^-(-1)$ yields:

$$c_3 = c\left[1 - \exp\left(-\frac{2}{\sigma}\int_{-1}^{0}\sqrt{r(x)}dx\right)\right]. \tag{5}$$

A further matching condition derives from the integration of Eq. (4) from $-1-\epsilon$ to $-1+\epsilon$:

$$\left.\frac{\partial p^+}{\partial \gamma}\right|_{\gamma=-1} - \left.\frac{\partial p^-}{\partial \gamma}\right|_{\gamma=-1} = \frac{j}{\sigma^2}.$$

The derivatives at -1 are given by:

$$\begin{aligned} \left.\frac{\partial p^+}{\partial \gamma}\right|_{\gamma=0} &= cr^{\frac{1}{4}}(-1)\left[-\frac{1}{\sigma} - \exp\left(-\frac{2}{\sigma}\int_{-1+\epsilon}^{0}\sqrt{r(x)}dx\right)\frac{\sqrt{r(-1)}}{\sigma}\right] \\ &= -cr^{\frac{1}{4}}(-1)\frac{1}{\sigma}\left[1 + \exp\left(-\frac{2}{\sigma}\int_{-1+\epsilon}^{0}\sqrt{r(x)}dx\right)\right], \end{aligned}$$

and

$$\left.\frac{\partial p^-}{\partial \gamma}\right|_{\gamma=-1} = c_3 r^{-\frac{1}{4}}(-1)\frac{\sqrt{r(-1)}}{\sigma}.$$

Inserting c_3 from Eq. (5) gives:

$$\left.\frac{\partial p^-}{\partial \gamma}\right|_{\gamma=-1} = cr^{\frac{1}{4}}(\gamma)\frac{1}{\sigma}\left[1 - \exp\left(-\frac{2}{\sigma}\int_{-1}^{0}\sqrt{r(x)}dx\right)\right].$$

Therefore we conclude that

$$-\frac{j}{\sigma^2} = -\frac{1}{2}cr^{\frac{1}{4}}(-1)\frac{1}{\sigma},$$

and thus

$$c = 2\frac{j}{\sigma} r^{-\frac{1}{4}}(-1).$$

It follows that

$$s = \frac{1}{2} r^{\frac{1}{4}}(0) r^{-\frac{1}{4}}(-1) \exp\left(-\frac{1}{\sigma}\int_{-1+\epsilon}^{0} \sqrt{r(x)}dx\right).$$

The σ-dependence for small σ stems from the exponential. Therefore

$$\log s \sim -\frac{1}{\sigma}\int_{-1+\epsilon}^{0} \sqrt{r(x)}dx.$$

We note that although the WKB approximation does not break down if r changes it sign in some domain of of γ, it gives there oscillating solutions, which are unphysical for probabilities or particle numbers. However, in this case our physical assumption on the possibility to pass to the stationary picture does not work anymore, since the diffusion is not able to stabilize the local explosive concentration growth, and the approach is inapplicable. Therefore, the reduction to the stationary picture is only reliable in the cases when the local proliferation rate is negative. It is interesting that the linear dependence of the mutation probability on σ^{-1} in the small world model survives also outside the applicability domain of the approximations done.

SI Appendix 2: Additional simulations: Influence of local/global connectivity

In this section we give details on the influence of the global connectivity (i.e., long-distance links) on the probability of a supercritical mutation. We note that the effect described here occurs additionally to the effect of long-distance links on mutations mediated by a larger percolation cluster as described in the main text. Furthermore it appears that it only occurs for the continuous mutation model and we found no evidence for it in the model with three discrete mutation states.

Construction of local networks with large cluster sizes. In Figs. S1 and S2 we compare the occurrence of supercritical mutations in the small-world network with those in a 2D lattice model. In order to achieve increases of the percolation cluster similar to those achieved in the small-world model, we used two network models in which connectivity is spatially restricted:

- Local model: We multiplied the function $\lambda(\gamma)$ with a constant prefactor C (with a cutoff of λ for values larger than 1.0). In this way, local connections are strengthened leading to larger clusters even if $\sigma = 0$.
- Next-nearest neighbor model: We added links to nodes that are next-nearest neighbors to a fraction of all nodes.

Note that in both cases it is possible to reach the percolation point simply by increasing the local connectivity, i.e. by effectively inceasing the strength of local links.

Simulations for continuous and discrete mutation landscapes. For each parameter set, we simulated for 1,000 to 10,000 times, calculated the average size of the infected cluster (at vanishing noise) and plotted it versus the fraction of runs with at least one mutation. Discussing the continuous case first, for $\sigma = 0.08$ we compare the fraction of runs with mutations for the three scenarios that increase the percolation clusters, Fig. S1. The cases with small-world links (increasing p to achieve larger clusters, circles) yield much higher fractions P_{mut} than the cases of local connectivity (crosses and squares) suggesting that it is not only the size of the cluster but also the network structure that is needed for the frequent occurrence of supercritical mutations.

Interestingly, for the case with three discrete muational states, we do not find the additional stochastic escape probablilty to the mutant state. Fig. S2 compares the next-nearest neighbor model (crosses) with the small-world model (circles), showing similar escape probablilty for similar mean cluster sizes for two different mutation rates χ. Additional research is needed to probe the cases with larger numbers of discrete steps.

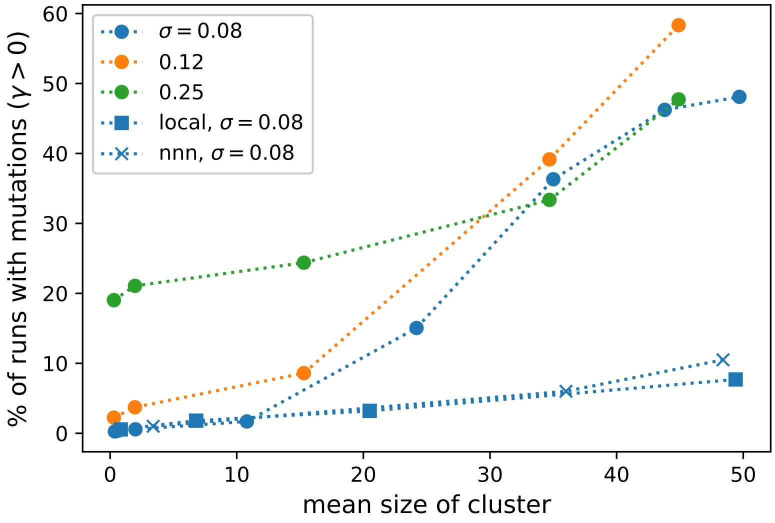

FIGURE S1. Fraction of runs with mutations for the simulations with continuous mutation space (nnn: next-nearest neighbors, see text for further explanation).

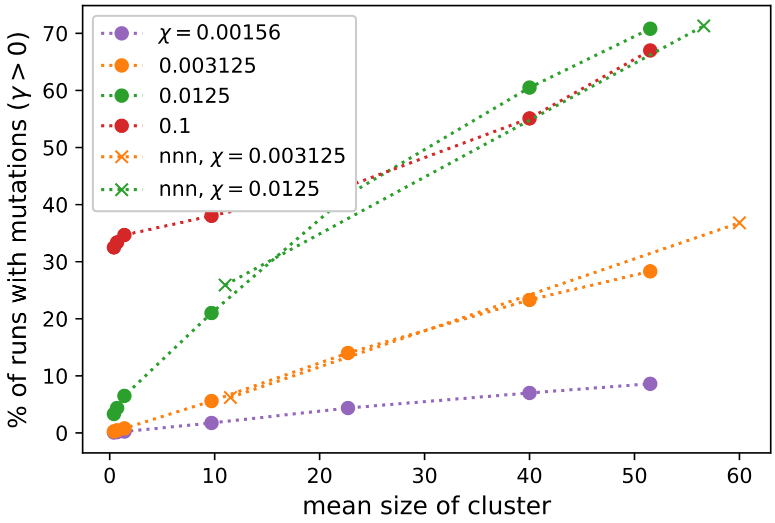

FIGURE S2. Fraction of runs with mutations for the simulations with discrete mutation space.
